# A Method to Measure Permeability of Permalloy in Extremely Weak Magnetic Field Based on Rayleigh Model

**DOI:** 10.3390/ma15207353

**Published:** 2022-10-20

**Authors:** Jinji Sun, Yan Lu, Lu Zhang, Yun Le, Xiuqi Zhao

**Affiliations:** 1 School of Instrumentation and Optoelectronic Engineering, Beihang University, Beijing 100191, China; 2 Ningbo Institute of Technology, Beihang University, Ningbo 315800, China; 3 Hangzhou Extremely Weak Magnetic Field Major Science and Technology Infrastructure Research Institute, Hangzhou 310000, China

**Keywords:** permalloy, extremely weak magnetic field, relative permeability, Rayleigh model

## Abstract

In order to solve the problem that the relative permeability of the permalloy is missing and difficult to measure accurately in an extremely weak magnetic field (EWMF, <1 nT), a method to measure the permeability in EWMF based on the Rayleigh model is proposed in this paper. In this method, the Rayleigh model for the magnetic material was first introduced. Then, the test system for measuring the permeability of permalloy for the standard ring specimen was set up. Based on the test data and the Rayleigh model, the functional expression applied to obtain the permeability in EWMF is achieved. Finally, the feasibility and accuracy of the method are verified by the permeability measurement of the custom large-size ring specimen in EWMF (<1 nT) and residual field measurement based on the four-layer shielding cylinder. This method can obtain the relative permeability in any EWMF and avoid test errors caused by extremely weak magnetization signals.

## 1. Introduction

The magnetically shielded room (MSR) used high permeable magnetic materials and high conductivity materials, such as permalloy and aluminum, to prevent the external magnetic field from entering its interior. Based on the principle of flux shunt [1,2,3] and eddy current loss [4,5], it can be used to provide an extremely weak magnetic field (EWMF) environment. In this environment, many frontier research studies for different kinds of fields can be carried out: for example, the measurement of weak signals such as magnetic heart and brain [6,7,8], the measurement of geophysical research samples [9], the measurement of electric dipole moments [10], the study of high-precision magnetic measuring instruments [11,12] and so on.

The MSR was first built by Bob J. Patton and John L. Fitch, who mainly used it for geophysical research [13]. Since then, many researchers around the world have successively developed the MSR, whose types include cuboid [14,15,16,17,18], cylinder [19] and spheroid-like [20,21]. The common feature of the existing MSR is the nested structure with multi-layer permalloy and aluminum, with the purpose of obtaining an EWMF environment (<1 nT). One of the most typical representatives is the Berlin Magnetically Shielded Room 2 (BMSR-2) built by the Physikalisch-Technische Bundesanstalt (PTB) [16]. BMSR-2 is composed of seven layers of permalloy and one layer of aluminum. With this design structure, the innermost layer of permalloy needs to work in the extremely low residual field: about 1 nT or even lower. Because the permeability of the permalloy varies nonlinearly with the applied magnetic field, it is necessary to obtain the permeability in EWMF. However, the permeability is generally measured at an applied magnetic field about 100 nT, which is considered to be the initial permeability. Using these data in the development of MSR could lead to a considerable overestimation of the shielding factor [22].

In order to solve this problem, Li et al. built an EWMF test system with a specialized large-sized ring specimen placed in a magnetic shielding cylinder, and the capability of the test system for the applied static magnetization field can reach as low as 4.5 × 10^−5^ A/m (≈0.057 nT) [22]. This method can directly obtain the desired permeability under EWMF, but the problems of the test errors and additional cost of time and money also exist due to the magnetic flux drift and the specialized large size ring and more turns of secondary windings (>400). Yamazaki et al. proposed a method of shielding effects (SEs) measurement for the multi-layer mu-metal cylinders and finite element analysis for the nonlinear model to obtain the incremental permeability of mu-metal in low magnetic fields [23]. In this method, a much more high-resolution magnetic field measurement sensor, such as a superconducting quantum interference device magnetometer and atomic magnetometer, is needed to achieve permeability in low magnetic fields, which limits its applied range due to the high-cost and special working environment of the sensors. British physicist Lord John Rayleigh [24] proposed that the parabolic dependence between the magnetic flux density of ferromagnetic materials and the magnetization field can be used to describe the hysteresis phenomenon of iron and steel under the low magnetizing fields (called the Rayleigh model). Since then, some scholars used the Rayleigh model to obtain the initial permeability of magnetic materials [25,26]. They established a shielding factor test system with Helmholtz coils applied for generating a 5 Hz and low-amplitude homogeneous magnetic field and finite element analysis applied for identifying the two important parameters in the Rayleigh model. These methods can be used to test the amplitude permeability of mu-metal thin foils less than 0.2 mm thick at 5 Hz, but the relative initial permeability at static magnetic fields cannot be obtained.

To obtain the data at an extremely weak static magnetic field, a method to measure the permeability of permalloy in EWMF based on the Rayleigh model is proposed. The method is divided into two processes: testing and identification. A ring specimen with standard size was used to obtain the magnetic characteristics of permalloy in the conventional magnetic field range. The identification process uses the Rayleigh model, fitting the test data in the Rayleigh region to obtain the initial permeability and the Rayleigh constant. The feasibility and accuracy of the proposed method were verified by building a permeability test system with an applied magnetic field less than 1 nT and a residual field test system with a four-layer permalloy cylinder. The proposed method can accurately obtain the relative permeability of permalloy in EWMF without the custom larger-size ring specimen, low magnetic field test environment and high-resolution sensor, and even the initial permeability when the magnetic field intensity tends to zero.

The arrangement of the remaining contents of this paper is as follows. In Section 2, the Rayleigh model for the magnetic material is first introduced. Then, the principle of the permeability measurement is presented. Test results and discussion are presented in Section 3. In Section 4, brief conclusions are summarized.

## 2. Principle of Permeability Measurement

### 2.1. Rayleigh Model for Magnetic Material

The model of the magnetization curve in a low magnetic field (Rayleigh region) proposed by Lord Rayleigh is shown in Equation (1), which is used to describe the nonlinear relationship between the magnetic flux density (*B*) and magnetic field strength (*H*)

(1) $$B=\mu_{0}\mu_{i}H+aH^{2},$$

where *µ*_0_ = 4π × 10^−7^ T·m/A is the permeability of vacuum; *µ*_i_ is the relative initial permeability of the magnetic material; *µ*_i_ = lim(*B*/*H*/*µ*_0_)(*H*→0), and *a* is the Rayleigh constant.

According to Equation (1) and the function between the magnetic flux density (*B*) and the magnetic field strength (*H*), *B* = *µµ*_0_*H*, the relative permeability of the permalloy, *µ*, can be expressed as

(2) $$\mu =\frac{B}{H\mu_{0}}=\mu_{i}+\frac{a}{\mu_{0}}H^{.}$$

Equation (2) shows that the relative permeability of the material increases linearly with *H* in the Rayleigh region, and the intercept is the initial permeability.

The two parameters, *µ*_i_ and *a*, are especially significant for the magnetization process of the Rayleigh region caused by the shift of the magnetic domain walls. The initial permeability *µ*_i_ is related to the reversible motion, which represents the linear increase in the magnetic flux density within the volume of the material under the influence of an increasing magnetizing field. The Rayleigh constant *a* is used to sketch nonlinear effects from the irreversible magnetization shift, which gradually covers the linear dependence with the increasing magnetizing field. The two parameters need to be identified by the experimental test and function fitting. Then, the *B*-*H* curves and the relative permeability curves can be obtained based on Equations (1) and (2), respectively.

### 2.2. The Measurement for Magnetic Material

**Figure 1:**
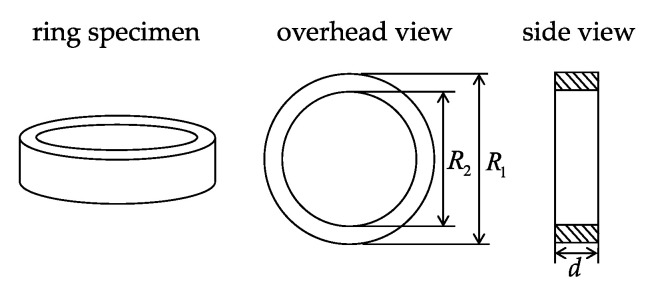
The overhead view and side view of the ring specimen.

In this paper, the simulated impact method was presented to test the *B*-*H* curves. During the test, the closed ring specimen with a primary winding and a secondary winding is used. The primary winding with direct-current (*I*_1_) injected is used to generate a magnetic field strength *H*, with a minimum value to be 0.01 mA. Based on the Ampere circuital theorem, the magnetic field strength (*H*) can be expressed as

(3) $$H=\frac{N_{1}I_{1}}{L_{e}},$$

where *N*_1_ is the turn number of the primary winding, and *L*_e_ is the effective magnetic circuit length. The secondary winding is used to detect flux linkage (ϕ) in the ring with a minimum value to be 0.1 *µ*Wb. According to the law of electromagnetic induction, the magnetic flux density (*B*) can be expressed as

(4) $$B=\frac{\phi }{N_{2}S_{e}},$$

where *N*_2_ is the turn number of the secondary winding, and *S*_e_ is the effective cross-sectional area. Before the experiment, *L*_e_ and *S*_e_ of the specimen can be calculated by using the size of the ring according to the SJ/T10281 standard, which can be expressed as

(5) $$L_{e}=\frac{C_{1}^{2}}{C_{2}},$$

(6) $$S_{e}=\frac{C_{1}}{C_{2}},$$

where *C*_1_ and *C*_2_ are the constants of the ring specimen and can be expressed as

(7) $$C_{1}=\frac{2\pi }{d}\ln{\left(\frac{R_{1}}{R_{2}}\right)}^{-1},$$

(8) $$C_{2}=\frac{4\pi \left(\frac{1}{R_{2}}-\frac{1}{R_{1}}\right)}{d^{2}}\ln{\left(\frac{R_{1}}{R_{2}}\right)}^{-3},$$

where *R*_1_, *R*_2_ and *d* are the outer diameter, inner diameter and thickness of the ring specimen, as shown in Figure 1, respectively. As the parameters are determined, the *B*-*H* curves can be calculated according to the applied current (*I*_1_) and the tested flux linkage (ϕ).

Equations (3)–(8) clearly show that the measurement range of the permeability is determined by the size of the ring specimen and the turns of the primary and secondary windings. To measure the *B*-*H* curve in the EWMF, we can need to increase the effective magnetic circuit length and reduce the turn of the primary winding to apply desired low magnetic field, and we also need to increase the effective cross-sectional area and the turns of the secondary winding to improve the detection signal of the magnetic flux.

## 3. Test Results and Discussion

### 3.1. The Test Method in EWMF Based on the Rayleigh Model

In order to obtain the data of an EWMF accurately and simply, two parts including the measurement process and identification process were performed. The measurement process provides relevant data for the identification process.

**Figure 2:**
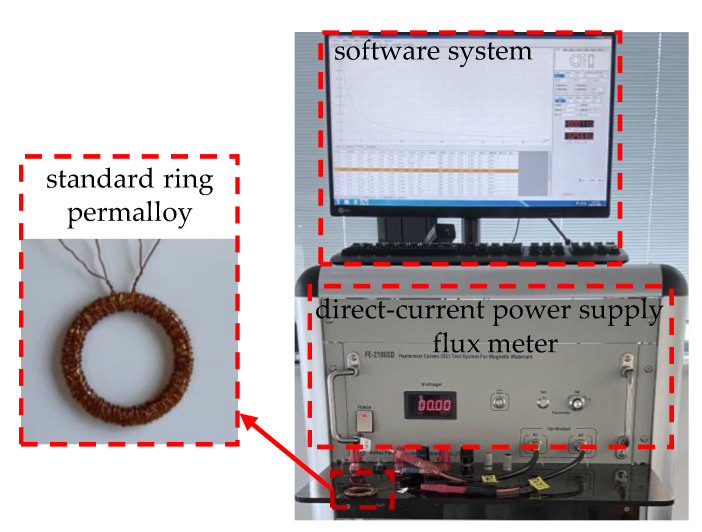
The outline of the common test system.

In the measurement process, the test system, mainly used to measure the *B*-*H* curve of permalloy, is first set up, as shown in Figure 2. The test system consists of a ring specimen with a primary winding and a secondary winding, test equipment with a direct-current power supply and a flux meter, and a software system. The test steps are performed as follows:

1. The dimensions of the ring specimen (outer diameter *A*, inner diameter *B*, and height *C*) were measured using vernier calipers.
2. The effective magnetic circuit length (*L*_e_) and cross-sectional area (*S*_e_) of the ring specimen were calculated according to the SJ/T10281 standard, and the turns of the primary and secondary windings were obtained according to the measured magnetic field range and Equations (3)–(8).
3. The primary and secondary windings are wound around the ring specimen. Notably, the high-temperature resistant tape should be used for insulation between windings and ring specimen, and the secondary winding should be wound before the primary winding.
4. The primary and secondary windings are connected to the power supply and the flux meter, respectively. The size of the measured ring, the turns of two windings, the range of magnetic field strength (*H* is set from 0.08 to 800 A/m) and other parameters are accurately set in the software. The value of the measured initial permeability corresponds to the one at the magnetic field strength of 0.08 A/m.
5. Based on Equations (3) and (4), the *B*-*H* curve, the permeability curve and the parameters including initial permeability (*µ*_i_) (*H* is 0.08 A/m) can be determined.

Then, the identification process is performed as follows. First, based on the measured *B*-*H* data in the Rayleigh region, the two key parameters, the initial permeability and the Rayleigh constant, can be identified by the function fitting. After that, extrapolating the measured data of the *B*-*H* curve according to the Rayleigh model with determined parameters, the *B*-*H* curve at an EWMF can be determined.

### 3.2. The Test Results and Discussion

(a) Test and parameter identification results for the Rayleigh model

**Figure 3:**
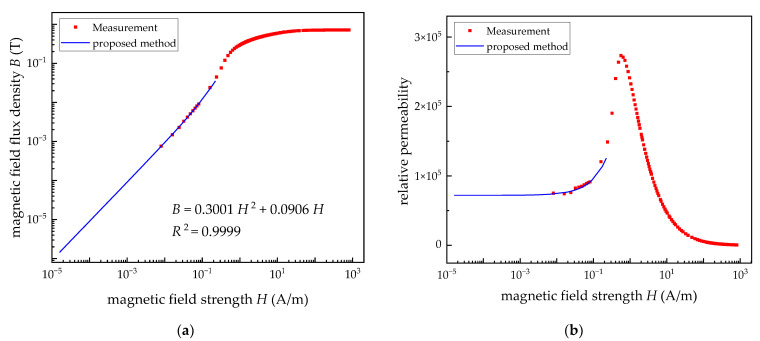
The comparison of the measurement and fitting curves. (**a**) The *B*-*H* curves. (**b**) The relative permeability curves.

In this work, 1J85, one of the permalloys with high permeability, provided by Beijing Beiye Functional Materials Corporation, was chosen. The parameters of the ring specimen are shown in Table 1, which are the commonly used parameters for the magnetic material test. The result for the *B*-*H* curve with *H* ranging from 0.008 to 800 A/m is shown in Figure 3a. Notably, the lower limit value of *H* is one order of magnitude lower than the set value, which is determined by the working principle of the test equipment. In addition, the curve related to the permeability and *H* was obtained by the first-order derivative of the *B*-*H* curve, which is shown in Figure 3b.

In order to determine Rayleigh constant *a* and the relative initial permeability *µ*_i_, the identification process related to the function fitting was performed based on the measurement data and Equation (1). The Rayleigh region should be lower than the coercivity (*H*_c_) for permalloy with high permeability. The fitting data were chosen to be in the range of 0.01 A/m to (0.5 × *H*_c_) A/m according to the previous studies for other soft magnetic materials in [27,28]. The fitting results are shown by the blue solid line in Figure 3, demonstrating a good agreement between the fitting curve and the test data. The fitting function is *B* = 0.3001*H*^2^ + 0.0906*H*. The Rayleigh constant is 0.3001, and the relative initial permeability is 72097, which is significantly lower than the measured relative permeability, being 91072 at 0.08 A/m. It can intuitively illustrate that the shielding effectiveness of the MSR can be overestimated using the permeability at 0.08 A/m.

(b) Verification for the proposed method

**Figure 4:**
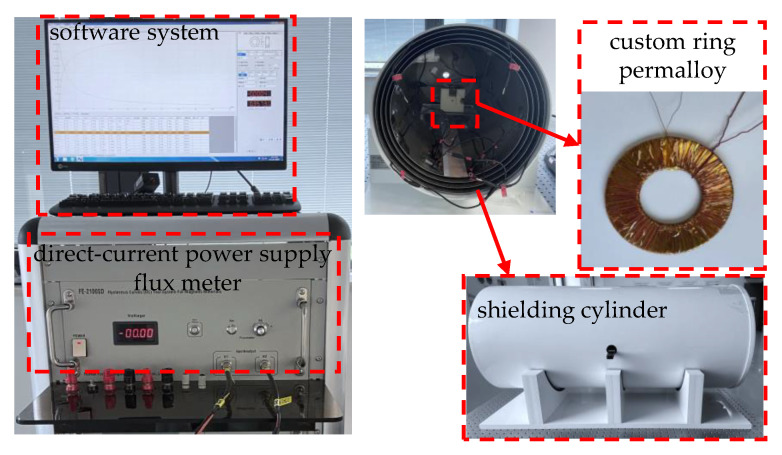
The outline of an extremely weak magnetic field (EWMF) test system.

Firstly, in order to verify the feasibility of the proposed method, an EWMF test system was built to measure the permeability of the permalloy in a low magnetic field, as shown in Figure 4. Different from the common test system, a custom large-size ring specimen and a four-layer magnetic shielding cylinder were introduced in the EWMF test system. The magnetic shielding cylinder is utilized to eliminate the interference of magnetostatic signals and extremely low-frequency magnetic fields in the geomagnetic environment. The custom ring specimen is used to measure the permeability in the EWMF, and the parameters are shown in Table 2. Based on the parameters of the custom ring specimen and Equations (3)–(8), the minimum of the magnetization field is calculated to be about 4.59 × 10^−5^ A/m, less than 1 nT, and the minimum of the measurable magnetic flux density is about 1 × 10^−8^ T, which is larger than the magnetic flux measurement resolution of the magnetic material testing instrument. Considering the accuracy and resolution of the test system, the permeability in the magnetic field strength from 8 × 10^−5^ A/m to 25 A/m was chosen. In addition, due to the testing magnetic flux signal being so small as an EWMF applied in permalloy that it is easily affected by geomagnetic field and other interfering magnetic fields, the tested ring specimen was put in the magnetic shielding cylinder with the residual field less than 1 nT as the measurement is performed. The procedure of the measurement in the EWMF is the same as that of the common test system except for the range of magnetic field strength (here, *H* is from 8 × 10^−5^ A/m to 25 A/m).

**Figure 5:**
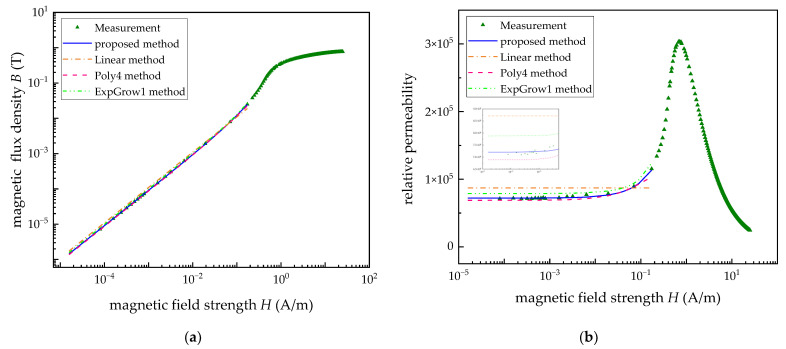
The comparison of four methods and the measurement data. (**a**) The *B*-*H* curves. (**b**) The relative permeability curves.

The measured results for the *B*-*H* curve and permeability curve of a custom large-size ring specimen in the EWMF are shown as the green dot line in Figure 5, which is consistent with the fitting curve of the Rayleigh model, as the blue solid line in Figure 5 shows. The measured minimum permeability at the *H* being 8 × 10^−5^A/m (0.1 nT) is 71,876, illustrating a 0.33% difference from the permeability (72,116) obtained by the fitting function of the Rayleigh model.

Secondly, to verify the optimal applicability of the Rayleigh model for the description of the relationship between the permeability and extremely weak magnetic field, the other three function models, including linear fitting (Linear method), 4th order polynomial fitting (Poly4 method) and one-phase exponential growth function (ExpGrow1 method), are selected for comparison. The comparison results are shown in Table 3 and Figure 5. As can be seen from the data in Table 3, compared with the other three models, the error between the fitted permeability and the measured value of the proposed method is the smallest. In addition, the curve obtained by the proposed method in Figure 5 is more consistent with the variation trend of the test data in the EWMF.

Finally, using the available four-layer magnetic shielding cylinder in our laboratory, the accuracy and validity of the Rayleigh model in estimating the shielding effect under EWMF were further analyzed and verified. The shielding cylinder shown in Figure 4 is composed of four layers of permalloy and one layer of aluminum, and its inner layer diameter and length are 36 cm and 90 cm, respectively. The thickness and layer spacing of the four-layer permalloy are the same as 1.6 mm and 1 cm, respectively. In this paper, finite element analysis was utilized to calculate the residual field in the center of the shielding cylinder. As the background magnetic field and *B*-*H* curve obtained by the Rayleigh model were set in the finite element model, the residual field in the center of the shielding cylinder was calculated to be 0.938 nT. While at the same position, the residual field was measured to be 0.987 nT after the shielding cylinder was degaussed, which has only 5% difference from the simulation. There is a good agreement between the simulation analysis and the actual measurement.

## 4. Conclusions

This paper proposes a method to measure the permeability of permalloy in EWMF based on the Rayleigh model. In this method, the magnetization and permeability curves were first obtained by measuring the ring permalloy of standard size. Then, two important parameters of the Rayleigh model, the relative initial permeability and the Rayleigh constant, were identified by function fitting the test data in the Rayleigh region. With the functional expression of the Rayleigh model, the measured data for the magnetization and permeability curves can be extrapolated to any desired weak magnetic fields or even zero magnetic fields.

The feasibility of the proposed method was verified by establishing an EWMF test system. The relative permeability of 8 × 10^−5^A/m, the lowest magnetic field that can be measured by EWMF test system, is only 0.33% different from that obtained by the proposed method. Furthermore, the accuracy of the proposed method was also verified by comparing the results between analysis and measurement with the existing four-layer magnetic shielding cylinder in the laboratory. Using the relative permeability obtained by the proposed method in the analysis, the central remanences obtained by the analysis and measurement have a 5% difference, showing a good agreement.

The method proposed in this paper for obtaining the permeability in EWMF is accurate and easy to carry out, without considering the custom large-size ring specimen, extremely weak magnetic test environment and high-resolution magnetic field measurement sensor. It also can be applied for the permeability measurement in EWMF of other soft magnetic materials. The method proposed mainly studies the magnetization curve and relative permeability curve of permalloy when the amplitude of the low magnetic field changes while its magnetic characteristics under varying magnetic fields are not involved in this paper.

## Figures and Tables

**Table 1:** Parameters of the tested standard ring specimen.

Symbol	Quantity	Standard Ring
*A*	outer diameter	40 mm
*B*	inner diameter	32 mm
*C*	Thickness	2 mm
*N*_1_	turns of the primary winding	60
*N*_2_	turns of the secondary winding	20

**Table 2:** Parameters of the tested custom ring specimen.

Symbol	Quantity	Custom Ring
*A*	outer diameter	100 mm
*B*	inner diameter	50 mm
*C*	Thickness	1 mm
*N*_1_	turns of the primary winding	1
*N*_2_	turns of the secondary winding	450

**Table 3:** Comparison of four methods and the measurement data.

Symbol	Fitting Function	*µ*_i_	*µ*_8 × 10^−5^ A/m_ *	Error Ratio *
Measurement	-	-	71,876	-
proposed method	*B* = 0.3001*H*^2^ + 0.0906*H*	72,097	72,116	0.33%
Linear method	*B* = 0.1095*H*	87,137	87,137	21.23%
Poly4 method	*B* = 3.5652*H*^4^ − 1.9207*H*^3^ + 0.4775*H*^2^ + 0.0865*H*	68,834	68,864	4.19%
ExpGrow1 method	*B* = −0.020188 + 0.01559$e^{\left(H+0.05273\right)/0.20399}$	78,757	78,787	8.77%

* The penultimate column in the table represents the relative permeability when *H* is 8 × 10^−5^ A/m. The last column in the table represents the error ratio between the measurement data and the permeability when *H* is 8 × 10^−5^ A/m.

## Data Availability

Not applicable.
